# Differential Expression of Insulin-Like Growth Factor-I Receptor on Human Bone Marrow-Derived Mesenchymal Stem Cells Induced by Tumor Necrosis Factor-α

**Published:** 2014-02-01

**Authors:** Z. Sahraean, M. Ayatollahi, R. Yaghobi, R. Ziaei

**Affiliations:** 1 *Department of Biology, Science and Research, Islamic Azad University, Fars, Iran*; 2 *Transplant Research Center, Shiraz University of Medical Sciences, Shiraz, Iran*

**Keywords:** Mesenchymal stem cells, Human bone marrow, Tumor necrosis factor-α, Insulin-like growth factor-I

## Abstract

Background: Cell-based therapy has been implicated in the treatment of liver diseases. Mesenchymal stem cells from various sources such as bone marrow are available. These cells are one of the major candidates in cell therapy. The production of insulin-like growth factor-I increases in the regenerating organ. The insulin-like growth factor-I in liver regeneration is effective after binding to insulin-like growth factor-I receptor.

Objective: To test our hypothesis that tumor necrosis factor-α can stimulate mesenchymal stem cells to express insulin-like growth factor-I receptor.

Methods: Bone marrow was aspirated from normal human donor after taking informed consent. Cells were isolated and cultured. Identification of cells was done by flowcytometry and functional tests. The fourth passage cells were treated with tumor necrosis factor-α at two doses of 1 and 10 ng/mL, and incubated for 2, 10, 24, and 48 hours. Insulin-like growth factor-I receptor gene expression was studied using real-time polymerase chain reaction.

Results: Flowcytometry showed that the human bone marrow mesenchymal stem cells were positive for CD90 and negative for CD45 and CD80. The insulin-like growth factor-I receptor gene expression was increased in tumor necrosis factor-α treated in comparison with untreated cells.

Conclusion: Treatment of human bone marrow-derived mesenchymal stem cells with tumor necrosis factor-α increases gene expression of insulin-like growth factor-I receptor. This finding may be used for increasing the effectiveness of stem cell therapy in those with acute hepatic failure.

## INTRODUCTION

Liver transplantation has become the treatment of choice for those with end-stage liver disease. Considering several limitations to access appropriate donors, stem cell-based therapies have gained importance in recent years. It has been shown that bone marrow (BM) is a source of stem cells for organ regeneration [1]. Bone marrow-derived mesenchymal stem cells (MSCs) have therapeutic potential in a wide range of diseases [2, 3]. Adult MSCs were first discovered by Friedenstein in 1966 [4]. The MSCs can differentiate into various tissues of mesodermal origin such as osteocytes, chondrocytes and adipocytes. They can also differentiate into endothelial cells [5].

Insulin-like growth factor-I (IGF-I) is a polypeptide with endocrine, paracrine, and autocrine effects [6]. Although many tissues secrete it, more than 90% of the circulating IGF-I is synthesized in the liver [7]. The production of IGF-I increases in regenerating organs. The IGF-I in organ regeneration, is effective after binding to IGF-I receptor (IGF-IR). The type I insulin-like growth factor receptor is a member of the tyrosine-kinase receptor superfamily involved in cell growth control, malignant transformation, and inhibition of apoptosis [8]. 

The regulation of organ regeneration has been studied extensively, and it has been shown to be dependent on the cytokines interleukin-6 and tumor necrosis factor-α (TNF-α) [9, 10]. TNF-α is a cytokine produced mainly by macrophages. TNF-α activates a wide array of cellular signaling pathways that result in divergent biological responses depending on the physiological setting [11]. It was reported that during liver regeneration after partial hepatectomy, increased TNF-α production initiates or promotes hepatocyte proliferation [12]. We conducted this study to test our hypothesis that the inflammatory factor TNF-α can stimulate human bone marrow-derived MSCs to express IGF-IR.

## MATERIALS AND METHODS

Culture and expansion of MSCs 

Bone marrow aspirates (3–5 mL) were obtained from the iliac crests of human donors aged between 17 and 30 years. They were donors of bone marrow to a related patient after obtaining approval of the Ethic Committee. Informed written consent was also obtained from the participants. The aspirates were diluted 1:1 with Dulbecco’s modified Eagle’s medium (DMEM)-low glucose (Gibco, USA) and layered over 3–5 mL of ficoll (Lymphoprep, Gibco, USA). The isolation method was according to a previously reported method [13] with some modifications. After centrifugation at 2000 rpm for 25 min, the mononuclear cells were removed from the interface by using a sampler. Cells were suspended in DMEM (including 10% fetal bovine serum) and centrifuged at 1300 rpm for 10 min and then resuspended in basal DMEM medium containing 10% fetal bovine serum (Gibco, USA), 1% penicillin (Gibco, USA), 1% streptomycin (Gibco, USA) and 1% L. glutamate (Gibco, USA). The cells were seeded at a density of 80,000/cm^2 ^in 25 cm^2 ^T-flasks and maintained at 37 °C in an atmosphere of 5% CO_2_. After 4–5 days of incubation, the non-adherent cells were removed and the medium was replaced every 3–4 days. In order to expand the MSC cells, the adhered monolayer was detached with trypsin-EDTA (Gibco, USA) for 5 min at 37 °C, after 14 days for the first passage and every 3–4 days for successive passages. During *in vitro* passage, the cells were seeded at a density of 5×10^3^ cells/cm^2^ and expanded for several passages until they no longer reached confluence. Each primary culture was replaced in three new flasks when MSCs grew to approximately 70%–80% confluence. Cells were frozen in the third and forth passages.

Flowcytometry 

Cell surface marker expression on MSCs was analyzed using a panel of antibodies. MSCs of the third passage at 70%–80% confluence were trypsinized with 0.25% trypsin-EDTA. The cell suspension was centrifuged at 1200×g for 5 min. The MSCs were diluted by wash buffer (49 mL phosphate buffered saline [PBS, Gibco, USA] and 1 mL FBS) to a concentration of 4×10^5^ cells/mL. The cell suspension was then centrifuged at 2100×g for 4 min at 4 °C. Subsequently, 100 μL of the cell suspension were added to each tube. The cells were stained for 30 min at 4 °C with fluorescent isothiocyanate (FITC)-conjugated CD45, CD80 and CD90 (all Dako, Denmark). An isotype control with FITC-labeled was included in each experiment. Negative control included non-stained cells and isotype-control stained cells. The labeled cells were thoroughly washed with wash buffer and analyzed on a flowcytometer (FACS Calibur Becton, Dickinson, USA).

Differentiation potential of MSCs 

The potential of the isolated cells to differentiate into osteogenic and adipogenic lineages was examined. For osteogenic differentiation, the fourth-passage cells were treated with osteogenic medium for three weeks. Osteogenic medium consisted of DMEM supplemented with 10^–8^ mol/L dexamethasone (Sigma-Aldrich, St. Louis, USA), 10 mol/L glycerol phosphate (Sigma-Aldrich, St. Louis, USA), 3.7 g/L sodium bicarbonate (Sigma-Aldrich, St. Louis, USA), and 0.05 g/L ascorbic acid (Sigma-Aldrich, St. Louis, USA). Osteogenesis was assessed by alizarin red staining. To induce adipogenic differentiation, the fourth-passage cells were treated with adipogenic medium for three weeks. Adipogenic medium consisted of DMEM supplemented with 1 mol/L hydrocortisone (Sigma-Aldrich, St. Louis, USA), 0.05 g/L ascorbic acid, 0.05 g/L indomethacin (Sigma-Aldrich, St. Louis, USA), and 10^–6^ mol/L dexamethasone.

Experimental groups 

MSCs of the fourth passage were trypsinized with 0.25% trypsin-EDTA and the cell suspension was centrifuged at 1200×g for 5 min. Then, the cells were treated with TNF-α at doses of 1 and 10 ng/mL, and incubated at for 2, 10, 24, and 48 hours. Differences in expression levels of IGF-IR were compared between TNF-α-treated and untreated MSCs. Also, differences in expression levels of IGF-IR were compared in different doses and times treatment by TNF-α.

IGF-IR gene expression 

MRNA extraction and cDNA synthesis

The expression levels of IGF-IR have been analyzed using the fourth passage of TNF-α-treated and untreated MSCs. The cell suspension (200 μL) was suspended in PBS. The total RNA was extracted from cultured cells incubating for 2, 10, 24 and 48 hours, in concentrations of TNF-α of 1 and 10 ng/mL, using RNX plus extraction kit (CinnaGen, Iran) according to the manufacturer’s instructions.

The cDNA was synthesized from RNA of MSCs in cDNA mix with total volume of 23 µL including 1 µL of random hexamers, 1 µL of dNTPs, 1 µL of Moloney murine leukemia virus reverse transcriptase (M-Mulv-RT), 0.65 µL of ribonuclease inhibitor, 7.35 µL of deionize water, and 2 µL of reverse transcriptase buffer on 10 µL of extracted RNAs. The thermocycling condition included one cycle at 42 °C for 90 min, and one cycle at 85 °C for 5 min.

Real Time-PCR

The relative expression of IGF-IR gene evaluated by Cyber Green based method. The primers of IGF-IR and β**-**actin genes designed by primer blast software of NCBI and evaluated by Oligo software (ver 6). The primer sequences and reaction conditions used in this study are listed in Tables 1 and 2, respectively. The quality of extracted RNA from different samples was evaluated by calculating the OD in a ratio of 260/280. The PCR mix for relative evaluation of the expression of IGF-IR gene with total volume of 20 μL included 0.4 μL of dye, 6 μL of deionized water, 0.8 μL of IGF-IR forward and reveres primers, 2 μL of template cDNA, and 10 μL of premix Ex Taq II (Takara BIO INC-Japan). Also, the PCR mix for relative evaluation of the expression of β**-**actin as housekeeping gene with total volume of 20 μL including 0.4 μL of dye, 6.8 μL of deionize water, 0.4 μL of β-actin primers, and 10 μL of premix of 2 μL of template cDNA.

**Table 1:** Primer sequences for real-time PCR of IGF-IR and ®-actin genes

Gene	Primer sequence		concentration
IGF-IR	Forward	5'-TCTGCCCGTCGCTGTCCTGT-3'	10 pm
	Reverse	5'-TCCCAAACGACCCCTGCCCA-3'	10 pm
β-actin	Forward	5'-GGGCGGCACCACCATGTACC-3'	10 pm
	Reverse	5'-GACGATGGAGGGGCCGACT-3'	10 pm

**Table 2:** The PCR thermocycling conditions for real-time PCR of IGF-IR and ®-actin genes

Cycle step	Temperature (°C)	Time (sec)
Initial denaturation	95	120
35 cycles Denaturation Annealing Extension	95 60.3 72	30 20 30

Statistical analysis 

A cycle threshold (Ct) value was calculated for each sample; sample values were averaged. The mean Ct value of target genes in each sample was normalized using β-actin gene Ct value to give a ∆Ct value. This was then normalized to control samples (∆∆Ct), and finally the 2^–∆∆Ct^. The SPSS ver 20 was used for data analysis. Kruskal-Wallis was used used for statistical analysis of the data.

## RESULTS

Characterization of isolated human MSCs 

**Figure 1:**
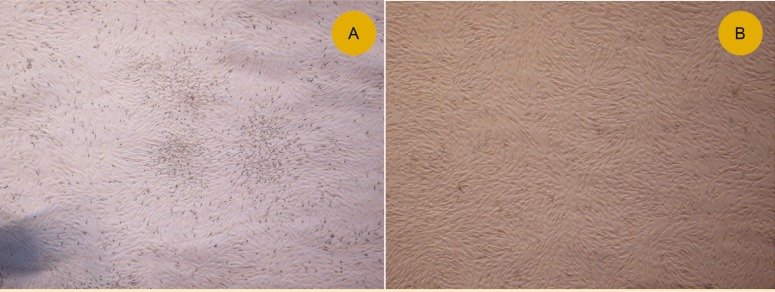
Isolation and culture of human bone marrow-derived MSCs. A) The presence of single cell-derived colonies composed of a few fibroblast-like cells; B) As the culture proceeded, the cells were spindled and wide-shaped fibroblastic morphology (4´).

When the cells reached a confluence of 75%–85%, the cellular morphology was imaged with an inverted microscope. The rapid expansion of the MSCs in culture was found to depend on the presence of single cell-derived colonies composed of a few fibroblast-like cells (Fig 1A). Bone marrow cells rapidly generated a confluent layer of cells possessing an elongated, fibroblastic shape (Fig 1B).

**Figure 2:**
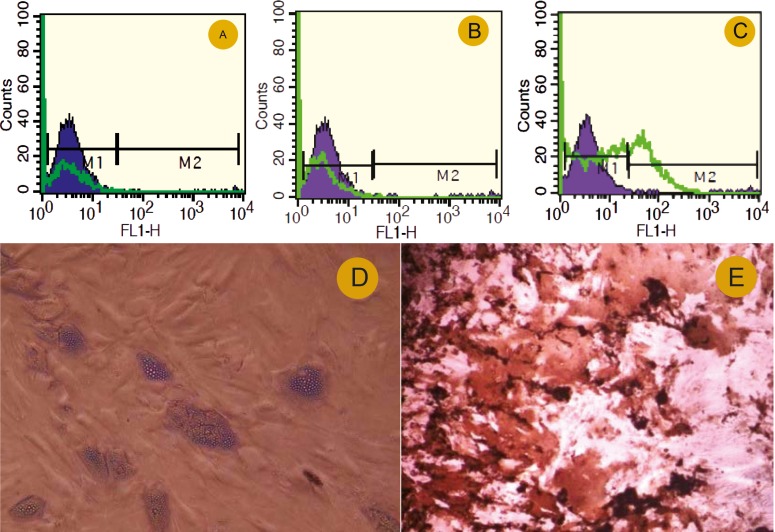
Characterization of the isolated human MSCs. Flowcytometry at the third passage demonstrated that the cells were negative for surface expression of CD45 and CD80 respectively (A and B); however they became positive for surface expression of CD90 (C). Isotype-control is displayed as black filled histogram and positive-stained cells as open histogram. D) The adipose droplet in differentiated cells after incubating with adipogenic media (10´); E) Osteogenic differentiation was positive for alizarin red staining (10´).

Flowcytometry showed that the human bone marrow MSCs were negative for surface expression of CD45 and CD80 (Fig 2A and 2B) and positive for surface expression of CD90 (Fig 2C). Ten-thousand events were acquired and analyzed by the Cell Quest software (BD Bioscience). Specific staining was measured from the cross point of the isotype with the specific antibody graph. 

MSCs are known to possess multi-lineage differentiation potential and can be directed to grow into specific cell lineages under certain micro-environmental conditions. The lipid vacuoles eventually combined and filled the cells. The accumulation of lipid in these vacuoles was assayed histologically (Fig 2D). Alizarin red staining confirmed the deposition of a mineralized extracellular matrix in the culture plates that could be detected after osteogenic differentiation (Fig 2E). These results indicated that the isolated cells have the basic properties of the MSCs.

TNF-α induce increased expression of IGF-IR mRNA in MSCs 

Expression of IGF-IR gene was increased in cells treated with 1 and 10 ng/mL of TNF-α compared with untreated cells. IGF-IR mRNA expression was induced in cultured bone marrow-derived MSCs based on TNF-α treatment in a concentration-dependent manner. These results indicated that the MSCs did not show the expression of IGF-IR gene without treatment with TNF-α.

**Figure 3:**
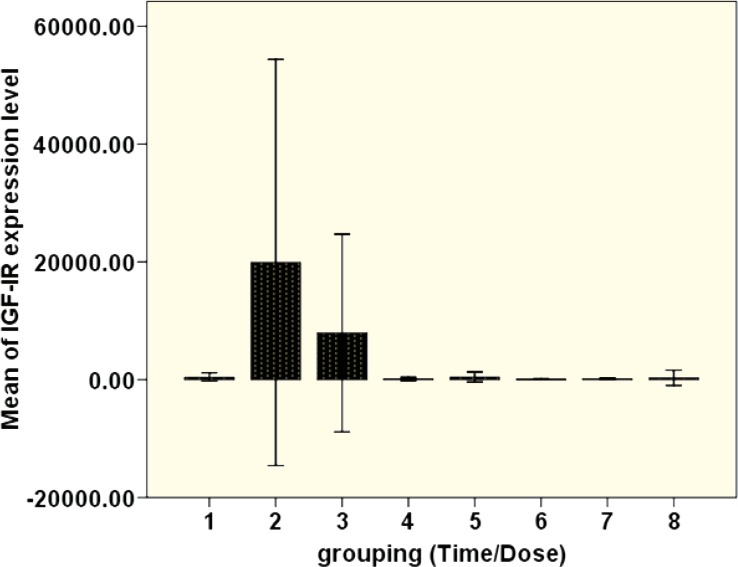
Differences in the mean of IGF-IR expression in the TNF-α-treated MSCs (p=0.03). Dose and time of treatment groups included 1) 1 ng/mL TNF-α for 2 hours; 2) 1 ng/mL TNF-α for 10 hours; 3) 1 ng/mL TNF-α for 24 hours; 4) 1 ng/mL TNF-α for 48 hours; 5) 10 ng/mL TNF-α for 2 hours; 6) 10 ng/mL TNF-α for 10 hours; 7) 10 ng/mL TNF-α for 24 hours; and 8) 10 ng/mL TNF-α for 48 hours

Our results indicated that maximum IGF-IR expression was found after 10 hours of treatment with 1 ng/mL TNF-α. However, a significant difference was observed in the 2-hour treatment with 10 ng/mL TNF-α in comparison to other time conditions. Also, the level of IGF-IR expression in treated MSCs with 1 ng/mL TNF-α was higher than in those treated MSCs with 10 ng/mL TNF-α in different conditioning times. There was a significant (p=0.03) difference between treated group with 1 ng/mL TNF-α in 10 hour and other treatments (Fig 3).

TNF-α enhanced the expression of the IGF-IR gene expression in both time- and dose-dependent manners. Treatment with 1 ng/mL TNF-α for 2, 10, 24, and 48 hours increased the expression of IGF-IR gene up to 256, 2048, 512, and 32 fold compared with untreated MSCs, respectively. Treatment with 10 ng/mL TNF-α for 2, 10, 24, and 48 hours increased expression of IGF-IR gene up to 128, 64, 32, and 22.6 fold compared with untreated MSCs, respectively.

## DISCUSSION

Bone marrow-derived MSCs have been explored as an important source for cell transplantation in clinic. The preclinical and clinical studies have provided evidence indicating that MSCs transplantations were beneficial in the treatment of a variety of diseases such as myocardial infarction, cerebral infarction, stroke, osteoarthritis, hepatic failure, and the complications of diabetes mellitus [14-16]. 

The MSCs are the cells with high reproducible characteristics and multipotential differentiation ability. They have been viewed as excellent candidates for cell therapy [17, 18]. Currently, many strategies have been developed to improve the migratory and homing capacity of MSCs. Among these strategies, *in vitro* induction of cultures, especially through their exposure to cytokines was studied. The feasibility for transplantation of primary or engineered MSCs has been demonstrated as cell-based therapy. The bone healing using MSCs might be improved with the use of some specific cytokines as the environmental factors in the cultured cells [19].

TNF-α is a major inflammatory cytokine. Hwang, *et al*, found that cytokines such as TNF-α can increase the sensitivity of MSCs to chemokines [20]. It has been shown that the expression of intercellular adhesion molecule-1 in endothelial cells increases in response to cytokines such as TNF-α [21].

Lieke, *et al*, observed up-regulation of interleukin-8 (IL-8) levels 8 and 24 hours after TNF-α stimulation in cord blood-derived multipotential MSCs as compared with unstimulated cells [22]. TNF-α was specifically selected to activate the classical nuclear factor-κB (NF-κB) signaling cascade of the innate immune system [23]. Henness, *et al*, have shown that TNF-α transcriptionally regulates IL-8 gene expression via transcription factor NF-κB [24]. 

In this project, we have investigated the expression level of IGF-IR in human bone marrow-derived MSCs treated with TNF-α in different doses and different incubation times in comparison with untreated cells.

The biological action of IGF-I is mediated through its cell surface receptors such as IGF-IR [25]. Following binding to phosphorylated IGF-I or insulin receptors, these docking proteins activate downstream signaling pathways including the mitogen-activated protein kinase and the phosphatidylinositol 3-kinase pathway whose activation leads to biological responses [26, 27].

In this study, we exposed the human MSCs to TNF-α, then analyzed the gene expression pattern of IGF-IR using real-time PCR. Our results indicated that TNF-α regulated the expressions of IGF-IR in a dose-response manner. We found that the optimal dose and incubation time for expression of IGF-IR on human marrow-derived MSCs was 1 ng/mL for 10 hour. *In vitro* induction of MSCs with TNF-α may be a useful strategy to enhance the therapeutic potentials of these cells for transplantation in clinical settings.

These findings may provide a piece of valuable information to improve human MSC capability for better therapeutic efficiencies of stem cell-based transplantation. Increase gene expression pattern of IGF-IR in human MSCs may be used for stem cell effectiveness before clinical stem cell therapy in acute liver failure.

## References

[1] Almeida G, Zanjani E, Porada Ch (2010). Bone marrow stem cells and liver regeneration. Experimental Hematology.

[2] Minguell J, Erices A (2006). Mesenchymal stem cells and the treatment of cardiac disease. Exp Biol Med..

[3] Mctaggart S, Atkinson K (2007). Mesenchymal stem cells: immunobiology and therapeutic potential in kidney disease. Nephrology.

[4] Friedenstein A, Piatetzky S, Petrakova K (1966). Osteogenesis in transplants of bone marrow cells. J Embryol Exp Morphol.

[5] Sato Y, Araki H, Kato J (2005). Human mesenchymal stem cells xenografted directly to rat liver are differentiated into human hepatocytes without fusion. Blood.

[6] Leroith D (1997). Insulin-like growth factors. N Engl J Med.

[7] Daughaday W, Rotwein P (1989). Insulin-like growth factors I and II: Peptide, messenger ribonucleic acid and gene structures, serum, and tissue concentrations. Endocr Rev.

[8] Valentinis B, Baserga R (2001). IGF-I receptor signaling in transformation and differentiation. Mol Pathology.

[9] Fausto N, Laird A, Webber E (1995). 1.Liver regeneration. 2. Role of growth factors and cytokines in hepatic regeneration. FASEB J.

[10] Cressman DE, Greenbaum LE, DeAngelis RA (1996). Liver failure and defective hepatocyte regeneration in interleukin-6-deficient mice. Science.

[11] Tracy K (1997). Tumor necrosis factor. Cytokine in health and disease.

[12] Yamada Y, Kirillova I, Peschon JJ, Fausto N (1997). Initiation of liver growth by tumor necrosis factor: deficient liver regeneration in mice lacking type I TNF receptor. Proc Natl Acad Sci.

[13] Ayatollahi M, Soleimani M, Geramizadeh B (2012). Conditions to improve expansion of human marrow-derived mesenchymal stem cells based on the rat samples. World J Stem Cells.

[14] Davatchi F, Abdollahi BS, Mohyeddin M (2011). Mesenchymal stem cell therapy for knee osteoarthritis. Int J Rheum Dis.

[15] Volarevic V, Arsenijevic N, Lukic M (2011). Mesenchymal stem cell treatment of the complications of diabetes mellitus. Stem Cells.

[16] Bao X, Wei J, Feng M (2011). Transplantation of human bone marrow-derived mesenchymal stem cells promotes behavioral recovery and endogenous neurogenesis after cerebral ischemia in rats. Brain Res.

[17] Jones E, gonagle D (2007). Human bone marrow mesenchymal stem cells in vivo. Rheumatology.

[18] Lundberg J, Le Blanc K, Söderman M (2009). Endovascular transplantation of stem cells to the injured rat CNS. Neuroradiology.

[19] Cheng H, Jiang W, Phillips FM (2003). Osteogenic activity of the fourteen types of human bone morphogenic proteins (BMPs). J Bone Surg Am..

[20] Hwang JH, Shim SS, Seok OS (2009). Comparison of cytokine expression in mesenchymal stem cells from human placenta, cord blood and bone marrow. J Korean Med Sci.

[21] Look DC, Rapp SR, Keller BT, Holtzman MJ (1992). Selective induction of intercellular adhesion molecule-1 by interferon gamma in human airway epithelial cells. Am J Physiol.

[22] van den Berk LC, Jansen BJ, Siebers-Vermeulen KG (2010). Mesenchymal stem cells respond to TNF but do not produce TNF. Journal of Leukocyte Biology.

[23] Bonizzi G, Karin M (2004). The two NF-kappaB activation pathways and their role in innate and adaptive immunity. Trends Immunol.

[24] Henness S, van Thoor E, Ge Q (2006). IL-17A acts via p38 MAPK to increase stability of TNF-α-induced IL-8 mRNA in human ASM. Am J Physiol Lung Cell Mol Physiol.

[25] Jones J, Clemmons D (1995). Insulin-like growth factors and their binding proteins. biological actions. Endocr Rev.

[26] Soos MA, Field CE, Siddle K (1993). Hybrid insulin/insulin-like growth factor-I receptors bind insulin-like growth factor-I but not insulin with high affinity. Biochem J.

[27] Treadway J, Frattali A, Pessin J (1992). Intramolecular subunit interactions between insulin and insulin-like growth factor-I ab half receptors induced by ligand and Mn/Mg ATP binding. Biochemistry..

